# There is a great variety of orthopaedic conditions presenting to a large hospital in Sierra Leone: a 3-year prospective study

**DOI:** 10.1007/s00590-022-03380-2

**Published:** 2022-10-14

**Authors:** Anthony Howard, William Bolton, Alexander Wood, Harry Hodgson, Julian Scott, David Jayne, Ibrahum Bundu

**Affiliations:** 1 NDORMS, Oxford University, Oxford, UK; 2 LIRMM, Academic Department, Leeds General Infirmary, University of Leeds, Leeds, UK; 3 Leeds Institute of Medical Research, St James, University of Leeds, Leeds, UK; 4 Vascular Institute, Leeds General Infirmary, Leeds, UK; 5 John Radcliffe Hospital, Oxford, UK; 6 Connaught Hospital, Freetown, Sierra Leone

**Keywords:** Sierra Leone, Orthopaedics, Trauma, Elective

## Abstract

**Purpose:**

In Sierra Leone there is a large void in orthopaedic research into the type of orthopaedic injuries, both acute and chronic. Improved data collection is essential in providing insight to guide health care planning and research. This study aims to outline the types of orthopaedic injury sustained.

**Method:**

Data were prospectively collected by local surgeons in the Orthopaedic outpatient department at a large hospital between January 2016 and January 2019.

**Results:**

The orthopaedic department saw a mean 728 patients per year, with mean age 24.0 years. The workload comprised of 64.92% acute orthopaedic conditions or their complications, with 35.08% elective orthopaedics. Fractures made up the largest proportion of clinical appointments, annually 244.33 fractures; however there was a high incidence of osteomyelitis.

**Conclusion:**

The study gives an important insight into the types and distribution of elective and trauma orthopaedic injuries sustained in Sierra Leone, which has not been previously reported, and highlights key areas where resources may be focused in order to improve clinical outcomes.

## Introduction

Developing countries bear the burden of 90% of the global motorised, industrial and conflict related injury [1, 2]. In African countries, injuries claim more lives than tuberculosis, malaria and HIV combined [3]. The mortality associated for example with road traffic injuries, in Africa, (28.3 per 100,000 population) is almost three times that when compared to Europe (11.0 per 100,000 population). In Sierra Leone, a developing African country, there is a large void in orthopaedic research into the type of orthopaedic injuries both acute and chronic. Improved data collection is essential in providing insight to guide health care planning and research [4]. The orthopaedic capacity in Sierra Leone is limited [5]. The largest burden of disease consequently falls on countries that are less able to cope, either financially or technically with injury [6, 7].

Sierra Leone is a developing country located on the West coast of Africa. The United Nations Development Programme in the 2018 Human Development Report, ranked Sierra Leone 184th out of 187, with an annual health spending at 18.3% of GDP [8]. The country has a population of 7 million and is one of the poorest countries in the world. Located in the capital of Sierra Leone, Freetown, is the Connaught Hospital, which is the only adult government hospital. Connaught Hospital was classified as tertiary, as described by Mulligan et al. [9], namely, a health facility which offers specialised care from highly skill personnel, possessing more sophisticated diagnostic techniques and advanced therapeutic technologies, and receiving referrals from district hospitals or other lower levels of care.

Previous work has attempted to estimate types of fractures through the use of various sampling techniques using questionaries’ [10]. Studies have estimated surgical capacity based on questionnaires from 10 West African Countries but not Sierra Leone, concluding that these countries were capital, equipment and personnel deficit [11]. The focus of other studies has been the etiology or mechanism of injury, which although may be helpful to identify preventive strategies, does not provide insight into managing the existing orthopaedic workload of tertiary hospitals at a micro level. The only previous study similar to our work in Sierra Leone was a respective analysis of the 2 months Accident & Emergency attendances and subsequent admission to the acute trauma ward in Connaught Hospital by Bundu et al. [12]. The study showed that head injury was the most common injury, followed by lower limb. This and other studies [13] have not identified detailed anatomical locations, merely body regions.

Other work has adopted a macro-level analysis. A census style study in Tanzania concluded that 508 patients daily were presenting to Hospitals with trauma-related complaints, with 300 patients suffering fractures [3]. Extrapolating this data in a simplified manner to Sierra Leone, which is smaller in size and population, this indicates a daily, approximately 58 patients presenting, 34 of these patient with fractures. Steward et al. [14] in a retrospective questionnaire study established that the incident of treated fractures in Sierra Leone was 570 per 100,000 persons. This paper intends to further explore the incidence and breath of orthopaedic conditions presenting to the orthopaedic services in Sierra Leone, and compare the distribution of elective versus trauma presentations.

## Methods

The data were prospectively collected in an Orthopaedic outpatient department at Connaught Hospital, Freetown, Sierra Leone, between January 2016 and January 2019.

### Sampling technique

Patients within the Outpatient setting were recruited consecutively; this approach ensured a good representation of the target population [15]. Patients were included if they were referred to the Outpatient Department by Accident and Emergency, referred to by another Hospital within Sierra Leone or being followed up after discharge from Connaught Hospital. Patients were excluded if they died whilst in hospital or those who failed to attend follow-up appointments following discharge.

No ethical approval was sought, as the audit department within the hospital approved the study as a clinical evaluation.

There remains controversy over the definitions of delayed and non-union in bone healing. Delayed and non-union was defined both on radiological and clinical findings. From the date of injury to excess of 3 months, without both radiological and clinical findings, consistent with bone union was defined as delayed union, and non-union defined as in excess of 6 months [16].

## Results

The clinic saw 2183 patients, (728 patients’ per year/61 patients’ per month), mean age 24.0 (2 months to 96 years) and 1:1.12 male to female ratio. The workload comprised of 64.92% acute orthopaedic conditions or their complications, with 35.08% elective orthopaedics.

As expected fractures made up the largest proportion of clinical appointments, annually 244.33 fractures and 14.67 dislocations presented. There is a high rate of osteomyelitis, soft tissue conditions and delayed/non/mal-union, Table 1. The anatomical location of the fractures was predominantly in the lower limb, Table 2.

**Table 1:** Break down of the types of elective and acute work in the outpatient department

Condition	Total number	Annual	Percentage
*Acute*			
Fractures	726	242.0	34.84
Osteomyelitis	154	51.33	6.96
Soft tissue	150	50.0	7.2
Mal or non-union	142	47.33	6.81
Vascular	48	16.0	2.3
Dislocation	44	14.67	2.11
Tendon or nerve	36	12.0	1.73
Head injury	29	9.67	1.39
Septic arthritis	28	9.33	1.49
*Elective*			
Spine	365	121.67	17.51
Osteoarthritis	265	88.33	12.72
Tumour	50	16.67	2.40
Rheumatoid arthritis	33	11.0	1.58
Congenital	20	6.67	0.96

**Table 2:** The anatomical location of fractures observed in the outpatient setting

Fracture site	Total	Annual
Femur	133	44.3
Tibia	109	36.3
Humerus	86	28.7
Ankle	69	23.0
Radius/ulna	41	13.7
Elbow	37	12.3
Radius	33	11.0
Skull	32	10.7
Hand	32	10.7
Clavicle	31	10.3
Spine	31	10.3
Foot	29	9.7
Pelvis	16	5.3
Femur/tibia	10	3.3
Patella	9	3.0
Rib	5	1.7
Ulna	4	1.3
Fibula	3	1.0
Tibia/fibula	2	0.7

The lower limb accounts for 50.57% of all osteomyelitis seen, with the humerus accounting for 13.06% and the forearm/hand totalling 8.52% of all cases seen within the OPD. The Clinic saw 10.33 cases per year of septic arthritis with the majority either in the knee (25.80%) or elbow/shoulder (32.26%). In soft tissue injuries, there were a large number of patients who presented with contractures, 9.33 patients per year. Delayed unions accounted for 12.69%, Non-unions 64.18% and Mal-unions account for 23.13% within the 134 seen in OPD over the three years. There is a far greater concentration of non- or mal-union in the upper limb, Table 3.

**Table 3:** The number of delayed, non-union and mal-union and their anatomical locations

	Delayed	Non-union	Mal-union
Ankle	2	3	0
Clavicle	1	13	1
Elbow	3	15	15
Femur	0	1	0
Foot	0	4	0
Hand	1	4	7
Humerus	0	3	2
Pelvis	0	3	2
Patella	0	10	0
Radius/ulna	1	12	10
Spine	9	14	1
Tibia	0	4	3
Total	17 (12.69%)	86 (64.18%)	31 (23.13%)

## Discussion

Little is known about the incidence and breath of orthopaedic conditions presenting in Sierra Leone, and there is a paucity of literature on the topic. This is the first study that reports actual as apposed to predicted numbers of fractures and the types of orthopaedic injuries over an extended period.

There are multiple limitations to this study. In particular it is retrospective in nature. Further, having only analysed the presentations to one hospital in the capital city it may not be reflective of the pathologies presenting elsewhere in the county, and there may be in particular great disparity between urban and rural locations.

The male to female ratio (1:1.12) in SL was different to other studies in Africa, where a higher male ratios where seen, such as 2:1 (male/female) and a previous study within SL where the risk of injury was lower in females [13]. This is thought to be due to a number of these studies were 40 years ago [17–20]. This highlights the need for recent epidemical studies, such as the current study, to be undertaken in order to understand if the picture between West African is different, or evolving, to changing social or industrial influences.

Within the acute patients seen the number of fractures seen in the outpatient setting, was comparatively lower compared to another study in Ghana, where the incident was 49.1% compared to 34.84% in Sierra Leone.

Similar to other study in Kenya [21] the highest proportion of injuries were to the lower limb, and the greatest number of fractures were to the femur, which is similar to other African countries, such as Tanzania [22] where the commonest cause was road traffic accidents in younger males. Whilst due to the limitations of the data collection, accident mechanism was not recorded consistently, however, anecdotally this accords with our experience in SL. We know from other African countries that the mortality associated with this type of fracture is in the region of 7.2% [23].

The incidence of Humerus fractures was higher than distal radius within the patient sample, which is contrary, to what has been observed in countries such as Uganda, Transkei and Nigeria. This comparative higher incident in the proximal compared to the distal upper limb is the polar opposite to countries such as England [24]. Antidotally, it is suspected that this relates different types of accident mechanism, however, this needs further research to see if preventative work safety information could reduce the incidence of this type of injury.

A previous study found that burns were the fourth most common reason for presentation to the Connaught Hospital, and these were particular common in children [12]. In our study, there were approximately 10 patients per year who were presented with contractures, secondary to burns, either directly or indirectly by the initial treatment of the bonesetters. The traditional bonesetters will rap the injured limb, typically a fracture, with a paste of herbs/chemicals then covering of fabric. It has been established in Nigeria that irrespective of age, sex, location, education status and financial position the bonesetter is the first port of call [25]. The use of this traditional fracture treatment is not confined to Africa and however is occurring in Asia, India and some western countries such as Turkey [26]. The resulting skin burns from this type of treatment can result in contractures and infections if the fracture is open. The World Health Organisation highlighted the practice of bonesetters and the potential irreversible complications, which relates to their methods and the delay to presentation to formal medical services [27].

The rates of delay, non- and mal-union, Table 3, non-union has the highest incident, and concentrated on the upper limb. Non-union fractures would arise from untreated fractures/late presented fractures and previously has been linked in Sierra Leone to the use of non-surgical management of fractures [28]. However, this will be a focus of future work, as this may be evidence of the effects of traditional bonesetter practice or the delay in presentation due to other reasons.

The high rates of infective conditions such as septic arthritis and osteomyelitis have enormous implications for these types of patients. The functional results of treating conditions such as septic arthritis late or where the cost of anti-bionics may prohibit complete eradications will be poor. Reducing function in a country without social support may condemn them to poverty, as their ability to work is reduced or curtained. Similarly, the effect of osteomyelitis may, without costly surgical management, result in the loss of limbs, or worse mean the patient becomes septic and dies.

The study shows that although the focus of previous studies has been on trauma, the elective demand for orthopaedic services is high, approximately 243 patients per year. In terms of the options available to treat conditions such as joint arthritis and common spinal conditions are limited. If patient symptoms of joint arthritis were not resolved by simple conservative measures, such as physiotherapy, weight loss and pain relieve joint replacement was not an option unless the individuals had the resources to seek surgery outside the country. There were 17 patients per year with different types of tumours, these ranges from benign to Osteosarcoma. In respect of elective/chronic problems, back pain and arthritis occupied the greatest; these proportions were similar to another study in Ghana [29].

## Conclusion

This is the first study in Sierra Leone that gives detailed analysis on the types of orthopaedic conditions presenting to a tertiary hospital in the capital Freetown. The study gives an important insight into the types of injuries not previously shown, but to where resource needs to be focused in order for better clinical outcomes. There are high rates of certain conditions such as non-union or mal-union that could be improved with greater surgical provision, which would provide better clinical outcomes.
